# Early responses to dehydration in contrasting wild *Arachis* species

**DOI:** 10.1371/journal.pone.0198191

**Published:** 2018-05-30

**Authors:** Christina Cleo Vinson, Ana Paula Zotta Mota, Thais Nicolini Oliveira, Larissa Arrais Guimaraes, Soraya Cristina Macedo Leal-Bertioli, Thomas Christopher Rhys Williams, Alexandre Lima Nepomuceno, Mario Alfredo Passos Saraiva, Ana Claudia Guerra Araujo, Patricia Messenberg Guimaraes, Ana C. M. Brasileiro

**Affiliations:** 1 Embrapa Recursos Genéticos e Biotecnologia, Parque Estação Biológica, CP, Final W5 Norte, Brasília, DF–Brazil; 2 Universidade de Brasília, Campus Darcy Ribeiro, Brasília, DF–Brazil; 3 Universidade Federal do Rio Grande do Sul, Campus do Vale, Porto Alegre, RS—Brazil; 4 Embrapa Soja, CP, Londrina, PR—Brazil; Louisiana State University College of Agriculture, UNITED STATES

## Abstract

Wild peanut relatives (*Arachis* spp.) are genetically diverse and were selected throughout evolution to a range of environments constituting, therefore, an important source of allelic diversity for abiotic stress tolerance. In particular, *A*. *duranensis* and *A*. *stenosperma*, the parents of the reference *Arachis* A-genome genetic map, show contrasting transpiration behavior under limited water conditions. This study aimed to build a comprehensive gene expression profile of these two wild species under dehydration stress caused by the withdrawal of hydroponic nutrient solution. For this purpose, roots of both genotypes were collected at seven time-points during the early stages of dehydration and used to construct cDNA paired-end libraries. Physiological analyses indicated initial differences in gas exchange parameters between the drought-tolerant genotype of *A*. *duranensis* and the drought-sensitive genotype of *A*. *stenosperma*. High-quality Illumina reads were mapped against the *A*. *duranensis* reference genome and resulted in the identification of 1,235 and 799 Differentially Expressed Genes (DEGs) that responded to the stress treatment in roots of *A*. *duranensis* and *A*. *stenosperma*, respectively. Further analysis, including functional annotation and identification of biological pathways represented by these DEGs confirmed the distinct gene expression behavior of the two contrasting *Arachis* species genotypes under dehydration stress. Some species-exclusive and common DEGs were then selected for qRT-PCR analysis, which corroborated the *in silico* expression profiling. These included genes coding for regulators and effectors involved in drought tolerance responses, such as activation of osmosensing molecular cascades, control of hormone and osmolyte content, and protection of macromolecules. This dataset of transcripts induced during the dehydration process in two wild *Arachis* genotypes constitute new tools for the understanding of the distinct gene regulation processes in these closely related species but with contrasting drought responsiveness. In addition, our findings provide insights into the nature of drought tolerance in wild germoplasm, which might be explored as novel sources of diversity and useful wild alleles to develop climate-resilient crop varieties.

## Introduction

Climate change models suggest a trend towards increasing risks in the agricultural sector worldwide, leading to the emergence of less productive or even inappropriate areas for cultivation in the tropics [1]. In addition, given the massive demand for crop irrigation, intense competition for freshwater is expected to develop and constitute a critical issue in the near future global bioeconomy. Research therefore needs to focus on plant adaptations to changing environmental constraints by developing technologies to increase crop productivity and sustainability in drought-prone areas [2,3]. Indeed, breeding programs are currently concerned with the adaptation of crops to the global climatic fluctuations by increasing their resilience to the impact of changes in rainfall distribution, heat stress intensification, and water scarcity.

Upon perception of a reduction in water availability, well adapted plants rapidly react to minimize water loss, maintain cellular osmotic adjustment, control water flux, and protect cells against oxidative stress and the damaging effects of dehydration. As part of this response, orchestrated molecular networks are activated, and interconnected responses at physiological, morphological, and biochemical levels are triggered to direct the plant to cope with water scarcity [4]. These molecular regulatory mechanisms include the production of osmoprotectants, antioxidants, and hormones, with abscisic acid (ABA) and ethylene (ET) acting as the principal signaling molecules that link transcriptional networks to environmental adaptation. Three signaling cascades are known to act following the onset of the drought stress [5–7]: (1) ABA-dependent signaling, mediated by the ABA-responsive element (ABRE) present in the promoter of various abiotic stress-related genes; (2) ABA-independent signaling towards the dehydration-responsive element (DRE); and (3) ethylene-signaling pathways that are associated with ERE (ethylene-responsive element) sequences in promoters.

Progress in genomics has facilitated the understanding of the mechanisms controlling abiotic stress responses in plants and led to the discovery of hundreds of genes involved in drought tolerance [6,8]. The generation of transgenic crops with enhanced drought tolerance is now a reality due to the use of such genes which activate or modulate specific pathways that improve plant ability to endure water-limited field conditions. However, the biological role of many drought-induced genes that participate in mechanisms underlying plant responses to water deficit is still unknown.

Peanut (*Arachis hypogaea*) is an important source of protein and oil and is, after soybean, the second most cultivated grain legume in the world, with an annual production of about 46 million tons [9]. A number of constraints affects peanut production, including susceptibility to certain biotic and abiotic stresses, and the breeding of improved cultivars has been hindered by its narrow genetic base [10–12]. Conversely, wild *Arachis* species have high genetic diversity and are mainly associated with the savannah-like Cerrado biome, where they have evolved mechanisms to adapt and survive under conditions of limited water availability [10,13]. Thus, the gene pool that involves wild *Arachis* species is a rich source of desirable agronomical traits, including drought tolerance, that could be used to expand the genetic basis of cultivated peanut through the introgression of wild genome segments [11,12,14]. The use of wild germplasm is one of the most promising alternatives to mobilize genetic variability, creating a strategic link between plant genetic resources and breeding programs.

The recent advances in *Arachis* genome sequencing [15,16] have opened new opportunities for the discovery of useful agronomic alleles harbored by wild germplasm. Indeed, transcriptome studies of wild *Arachis* made possible the identification of candidate genes responsive to several biotic and abiotic stresses, including root-knot nematode attack, fungal infection, and drought, to be deployed in peanut breeding programs[12,17–22]. More recently, the global gene expression profiling of wild *A*. *duranensis* and *A*. *magna* subjected to gradual water deficit enabled the identification of candidate genes associated with major processes underlying plant tolerance to drought conditions, such as those involved in signal transduction, primary metabolism, hormone homeostasis, and protection/adaptation of cellular structures [19,23].

In the present study, a comprehensive transcriptome characterization of two accessions of wild *Arachis* species (*A*. *duranensis* and *A*. *stenosperma*), contrasting in their transpiration behavior during water deficit imposition [24], was conducted aiming to improve the current understanding of the mechanisms deployed by these genotypes to withstand water deficit. *A*. *duranensis* and *A*. *stenosperma* are also the parents of the mapping populations used to construct the diploid genetic map for the AA genome of *Arachis* [25], facilitating their future use in breeding programs. Comparative transcriptome studies in plants have profited from the vast amount of genomic information produced in recent years to compare gene expression by a developmental approach in a single species or by an evolutionary approach across multiple species [26]. Herein, a comparative transcriptomic analysis of these two contrasting *Arachis* genotypes subjected to dehydration revealed a number of common and exclusively regulated genes coding for regulators and effectors involved in water deficit responses. Further qRT-PCR validation revealed promising candidate genes for drought tolerance in wild germoplasm.

## Materials and methods

### Plant material and dehydration treatment

Seeds of *Arachis duranensis* (accession K7988) and *A*. *stenosperma* (accession V10309) were obtained from the Brazilian Arachis Active Germplasm Bank, maintained at Embrapa Genetic Resources and Biotechnology (Brasilia, Brazil). Seeds were germinated on germitex paper with 2% (w/v) Ethrel (2-chloroethylphosphonic acid) and 0.05% (w/v) Thiram (tetramethylthiuram disulfide), at 25 ± 1°C and 65 ± 5% relative humidity. Fifteen-day-old seedlings were transferred to a hydroponic system in a randomized block experimental design, with 30 individual biological triplicates for *Arachis duranensis* and 30 for *A*. *stenosperma*. Individuals were placed in polystyrene supports (S1 Fig) that allowed seedling roots to be completely immersed in the aerated pH 6.6 balanced nutrient solution [27]. Plantlets were grown in a controlled environment chamber, at 25 ± 2°C and 60 ± 5% relative humidity, under a photosynthetic photon flux density of 1.5 x 10^3^ μmoles m^-2^ s^-1^, equivalent to 8.93 x 10^4^ lux, and a 12 h day length, as described previously [27]. After four weeks, the dehydration treatment was initiated by withdrawing the nutrient solution (air-drying on chamber temperature; S1 Fig), and roots for both genotypes were collected after zero (T0 or control); 25 (T25); 50 (T50); 75 (T75); 100 (T100); 125 (T125) and 150 (T150) min. Four root samples were pooled per biological replicate for each time point, immediately frozen in liquid nitrogen, and stored at -80°C until RNA extraction.

### Physiological analysis

The photosynthetic rate (*A*), stomatal conductance (*g*_*s*_), transpiration rate (TR), vapor pressure deficit based on leaf temperature (VpdL), intercellular carbon dioxide (CO_2_) concentration (*C*_*i*_), and leaf temperature, were measured as previously described [28], using a portable Photosynthesis System (LI-COR, model LI-6400) set at a light intensity of 1,000 μmol m^-2^ s^-1^. Data were collected at the first three time points (T0; T25 and T50), corresponding to the control and the first 50 min of dehydration from the first three quadrifoliate leaves. Measurements were made on the middle leaflet of completely expanded leaves. Data obtained for each genotype were compared using the t-test (p ≤ 0.05).

### cDNA library sequencing and *in silico* expression profiling

Root total RNA extraction, purification, and integrity checking were performed according to [29]. For cDNA library construction, RNA samples were pooled for each genotype: a control group (CTR), formed from samples collected at T0, and a stressed group (STR), formed from samples collected throughout the dehydration treatment (T25 to T150) and pooled in equal amounts. cDNA library construction and high-throughput sequencing were conducted at Fasteris SA (Plan-les-Ouates, Switzerland), according to the Illumina HiSeq2000 protocol (paired-end 2x100 bp).

The Illumina raw reads were trimmed using the Trimmomatic software version 0.33 [30] and the quality checked using FastQC (http://www.bioinformatics.babraham.ac.uk/projects/fastqc). For mapping to the reference genome, the high-quality reads were submitted to the default settings of the GMAP/GSNAP package [31] against the annotated *A*. *duranensis* genome available at PeanutBase (http://peanutbase.org/). The mapped reads were counted using the HTSeqCount software version 0.9.1 [32] using the resolution mode union and the analysis of differential gene expression between CTR and STR samples was conducted using the edgeR package in R [33]. Mapped genes were considered as significantly Differentially Expressed Genes (DEGs) when their relative gene expression levels had an adjusted *p-value* (false discovery rate; FDR) <0.05 and at least 4-fold change (FC) value between STR and CTR samples (log_2_FC > 2 or < -2). The distribution of the DEGs in the *Arachis duranensis* chromosomes (http://peanutbase.org/) was made using the Circa software (http://omgenomics.com/circa/). Volcano analysis was conducted using the Trinity Differential Expression scripts (https://github.com/trinityrnaseq/trinityrnaseq/wiki/Trinity-Differential-Expression).

### Functional analysis

For the comparison with other legumes species, the OrthoFinder algorithm [34] was used to analyze the complete proteome of four legume species (*Arachis duranensis*; *Arachis ipaënsis*; *Glycine max*; and *Phaseolus vulgaris*). OrthoFinder performs a blast all-against-all and uses the reciprocal best hit for each species to cluster the Ortholog Groups (OGs). Family-companion (https://bbric-pipelines.toulouse.inra.fr/family-companion) was used to generate the binary matrix of the OGs, which was submitted to UpSetR [35] for comparison visualization.

The functional annotation of *A*. *duranensis* (http://peanutbase.org/) gene models was used to classify the DEGs identified in both *Arachis* species for the Gene Ontology (GO) and the Transcription Factors (TFs). The Hypergeometric Test for overrepresentation from the FUNC package [36] was used to test for enriched GO categories among the DEGs. Genes that were differentially expressed were coded “1”, whereas mapped genes received the code “0”. The results were then analyzed by the REVIGO method to decrease redundancy [37]. DEGs coding for putative TFs were classified according to their respective family according to the Plant TF database (http://planttfdb.cbi.pku.edu.cn/).

To assign functional terms to candidate genes, sequences of all *A*. *stenosperma* and *A*. *duranensis* DEGs were submitted to the Mercator software (http://mapman.gabipd.org/web/guest/app/Mercator) against the *Arabidopsis thaliana* reference database, using the default settings. The mapping file predicted by Mercator was further used as input for the MapMan software (http://mapman.gabipd.org/) for visualization of the gene expression data from the two *Arachis* species in different abiotic stress pathways.

### qRT-PCR analysis

Expression profile of selected DEGs was conducted by quantitative reverse transcription PCR (qRT-PCR) analysis using RNA samples from roots of *A*. *duranensis* and *A*. *stenosperma* collected at each time-point (T0 to T150). Total RNA was treated with DNase and converted into cDNA, as previously described [29], and used as the template for qRT-PCR reactions carried out on a StepOne Plus Real-Time PCR System (Applied Biosystems, Foster City, USA). Specific primers for the 20 selected DEGs (S1 Table) were designed using previously described parameters [29], and qRT-PCR reactions were conducted in technical triplicates for each sample, using No Template (NTC) and No Amplification (NAC) as negative controls. Average cycle threshold (Cq) values were estimated using the online real-time PCR Miner tool [38] and normalized with two reference genes (ACT1 and UBI2), in accordance with [39]. For a first series of qRT-PCR analysis, expression ratios of DEG transcripts were validated using STR and CTR sample pools, as described above for cDNA library construction. A more detailed qRT-PCR analysis was then conducted to determine the expression ratios of 15 DEGs at each of the six stressed points (T25 to T150) relative to control point (T0), in independent biological triplicates. The relative quantification of transcripts was determined and statistically tested using the REST 2009 v. 2.0.13 software [40].

## Results

### Wild *Arachis* physiological response to dehydration stress

**Fig 1:**
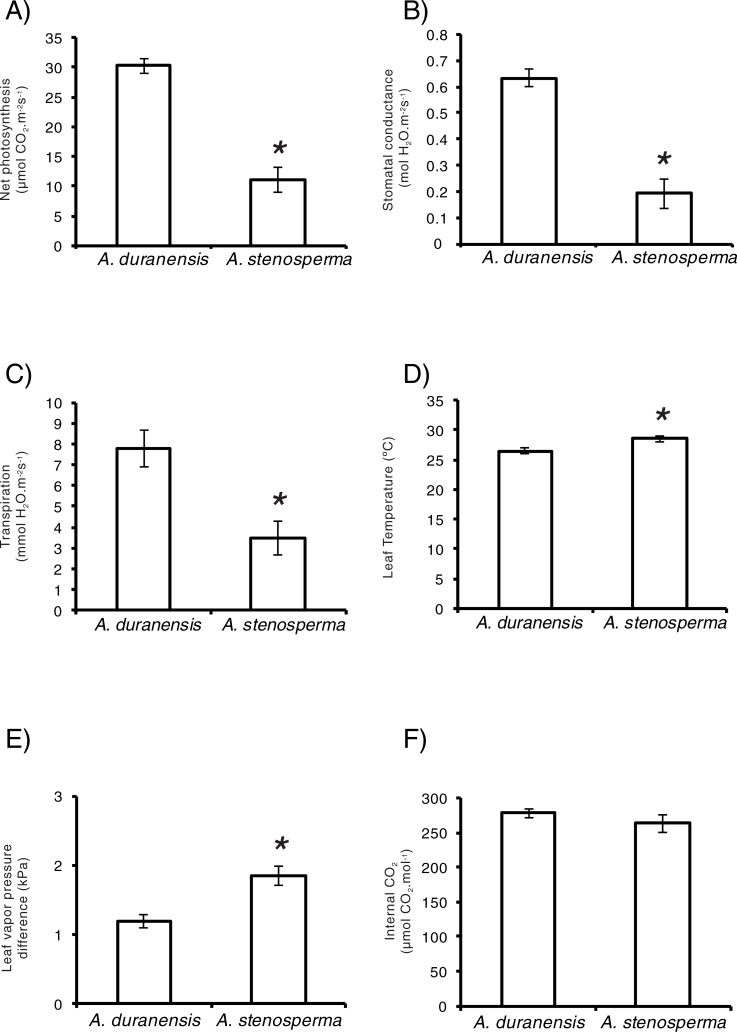
Physiological analysis of *Arachis* spp. plants during the early stages of dehydration. A) Photosynthetic rate; B) Stomatal conductance; C) Transpiration rate; D) Leaf temperature; E) Leaf vapor pressure deficit; and F) Internal CO_2_ concentration of *A*. *duranensis* and *A*. *stenosperma* plants subjected to dehydration. Data represent averages calculated over the early stages (0, 25 and 50 min) of the drought imposition and standard error. Statistically significant differences (n = 3, Student’s t-test, p<0.05) are indicated by asterisk.

Physiological analyses were carried out at the beginning of the experiment (0, 25, and 50 min) in order to compare the two wild *Arachis* genotypes (Fig 1; S2 Table). *A*. *duranensis* showed a higher photosynthetic rate, stomatal conductance, and transpiration rate, together with a lower leaf temperature, and vapor pressure difference than *A*. *stenosperma* (Fig 1). The intercellular CO_2_ concentration was the only physiological parameter for which no statistically significant difference between the two species was observed (Fig 1F). Any changes in these parameters were detected over the course of the experiment (data not shown), most likely due to the fact that these measurements were made at the beginning of the stress treatment which, while a long enough period to introduce a pronounced stress in roots of these species, as demonstrated by the alterations in gene expression presented below, was not long enough to affect leaf gas exchange [27]. Importantly, previous work with the same accessions of these species showed a more conservative whole plant transpiration response to water deficit in *A*. *duranensis* compared to *A*. *stenosperma* [24]. On this basis, the genotypes of *A*. *duranensis* and *A*. *stenosperma* used in this study will refer as ‘tolerant’ and ‘sensitive’, respectively, a designation supported by the gene expression results presented below.

### Wild *Arachis* transcriptome response to dehydration stress

Illumina HiSeq2000 sequencing of *A*. *duranensis* and *A*. *stenosperma* transcriptome libraries generated a total of 17.5 and 24.8 million raw reads (Table 1). After removing adapters and low base quality reads, more than 94% high-quality reads (≥ Q30) were used for downstream analyses. The sequencing data was deposited in the NCBI-SRA database under the BioProject number PRJNA284674.

**Table 1:** Summary of sequencing data. Illumina HiSeq2000 sequencing data of *Arachis* spp. transcriptome and mapping of reads to the reference *A*. *duranensis* genome.

Libraries	AdCTR	AdSTR	AsCTR	AsSTR
**Yield (Mb)**	1,817	1,691	2,444	2,518
**%PF**^**a**^	97.10	96.90	97.61	96.79
**Total number of reads**	9,085,802	8,453,190	12,219,081	12,587,737
**≥ Q30(%)**^**b**^	96.08	95.97	94.87	94.88
**Mapping in the reference**	***A*. *duranensis***		***A*. *stenosperma***	
**Total number of mapped gene models**	21,125		21,502	
**Significantly expressed genes**^**c**^	1,257		1,725	
**Differentially expressed genes (DEGs)**^**d**^	1,235		799	

^a^ PF stands for “passed filter” i.e. clusters that fulfill the default Illumina quality criteria.

^b^ A Q score of 30 (Q30) means that there is a 1 in 1,000 chance that base call is wrong.

^c^ FDR< 0.05

^d^ FDR< 0.05 and Log2FC (> 2 or < -2).

High-quality reads of both species were mapped onto the reference *A*. *duranensis* genome (http://peanutbase.org/) to allow a comparative transcriptome analysis of their water deficit responsiveness. The majority of the reads from roots of *A*. *duranensis* (98.71%) and *A*. *stenosperma* (95.46%) could be mapped to the reference genome, respectively resulting in the identification of 21,125 and 21,502 gene models (Table 1; S3 and S4 Tables), from the 36,734 predicted protein-coding genes previously delineated for *A*. *duranensis* [15].

**Fig 2:**
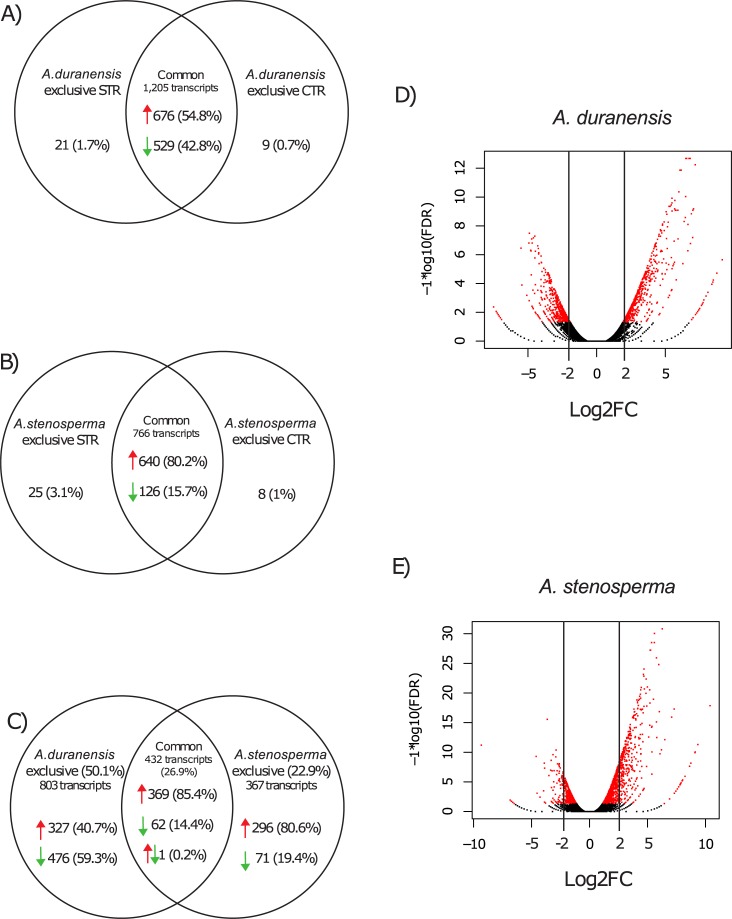
Expression patterns of *Arachis* spp. transcripts in response to dehydration. Venn diagrams showing the number of common and exclusive Differentially Expressed Genes (DEGs) in STR and CTR libraries of *A*. *duranensis* (A); *A*. *stenosperma* (B); and in both species (C). STR indicates the library from stressed (T25 to T150) samples and CTR the library from control (T0) samples. Red arrows indicate DEGs significantly upregulated during dehydration and green the downregulated DEGs. Volcano plots showing the comparison of gene expression profiles between STR and CTR samples in *A*. *duranensis* (D) and *A*. *stenosperma* (E), with each gene represented by one dot (DEGs in red and not significant in black).

*In silico* analysis of differential gene expression, using only FDR< 0.05 as parameter, 1,257 genes could be assigned as significantly expressed between the two *A*. *duranensis* libraries (AdSTR and AdCTR; Table 1). A similar number (1,725) of significantly expressed genes was also identified between the two *A*. *stenosperma* libraries (AsSTR and AsCTR; Table 1). When the fold change parameter (log_2_FC > 2 or < -2) was applied to select among them the Differentially Expressed Genes (DEGs), a total of 1,235 DEGs was assigned to *A*. *duranensis* and 799 to *A*. *stenosperma* (Table 1; S5 and S6 Tables). In *A*. *duranensis* (Fig 2A; S5 Table), the great majority of DEGs (1,205; 97.6%) were present in both STR and CTR libraries, with 676 upregulated and 529 downregulated in response to dehydration, whereas only 21 (1.7%) were exclusive to the STR library and nine (0.7%) to the CTR library. Likewise, *A*. *stenosperma* STR and CTR libraries shared a total of 766 DEGs, with the majority (640) being positively regulated, while 25 DEGs were exclusive to the STR library and eight to the CTR library (Fig 2B; S6 Table).

Comparing the overall responses to dehydration of the two species, a total of 1,602 DEGs were identified in at least one of the species (Fig 2C; S7 Table), with 432 being commonly regulated in both *A*. *duranensis* and *A*. *stenosperma* during the dehydration treatment. The majority of common DEGs were induced (369) whilst fewer were repressed (62). Only one DEG, coding for a putative SENESCENCE-ASSOCIATED PROTEIN (SAP; S1 Table). displayed contrasting regulation between the species (induced in *A*. *duranensis* and repressed in *A*. *stenosperma*). Regarding the exclusive DEGs, *A*. *duranensis* showed a higher number (803; 50.1%) than *A*. *stenosperma* (367; 22.9%) (Fig 2C; S7 Table). The number of upregulated *A*. *duranensis*-specific DEGs (327) is lower than those downregulated (476) whereas the number of upregulated *A*. *stenosperma*-specific (296) DEGs clearly exceeded that of those downregulated (71; Fig 2C; S7 Table). These results suggested that most of the genes responsive to the dehydration treatment were species-specific and that the drought responsiveness in the tolerant *Arachis* genotypes was associated with the modulation of two-times more specific transcripts when compared to the contrasting sensitive genotype.

Gene expression profiles *in silico* represented by volcano plots (Fig 2D and 2E) support the idea that the accessions of the two *Arachis* species had a different pattern of expression under dehydration stress. Despite the similar number (1,257 genes for *A*. *duranensis* and 1,725 for *A*. *stenosperma*; Table 1) of significantly expressed genes (FDR< 0.05), after application of the fold change parameter (Log_2_FC > 2 or < -2), it drastically decreased to half for *A*. *stenosperma* (799 genes) whereas it was almost the same for *A*. *duranensis* (1,235 genes; Table 1; Fig 2D and 2E). Besides to have 1.5-times as many DEGs, the overall expression magnitude of genes in response to dehydration is greater in the tolerant genotype than in the sensitive, in particular for downregulated genes.

**Fig 3:**
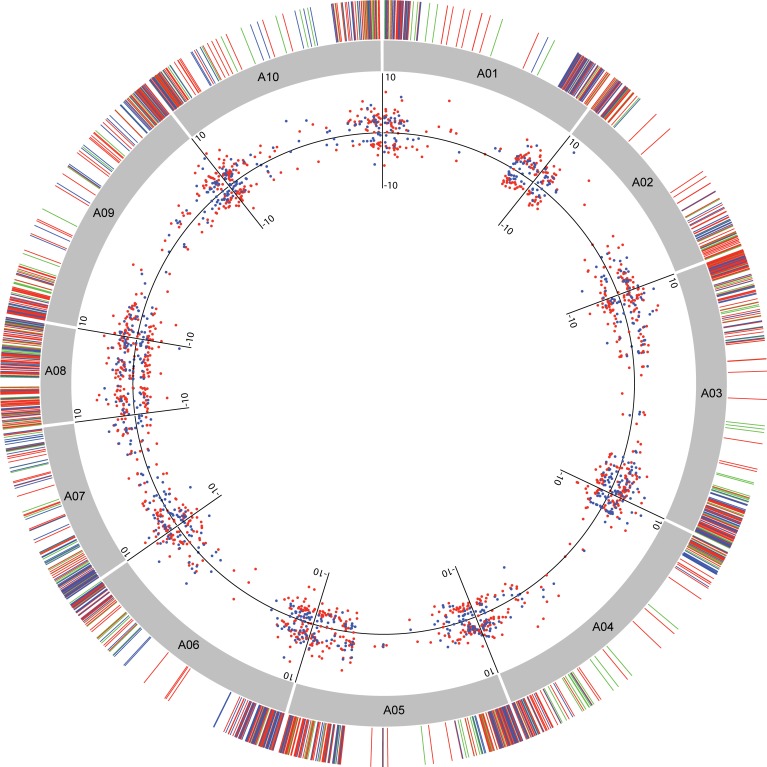
Circos plot detailing chromosome distribution of DEGs. Distribution of the 1,602 DEGs in the ten chromosomes of *A*. *duranensis* (A01 to A10). The outer lines represent exclusive *A*. *duranensis* (red) and *A*. *stenosperma* (blue) and common (green) DEGs. The inner dots represent the distribution of Log2FC values for each up- and downregulated DEG in *A*. *duranensis* (red) and *A*. *stenosperma* (blue), with the line indicating Log2FC = 0.

The chromosomal distribution analysis of the 1,602 DEGs identified in at least one of the species revealed an uneven distribution across all ten chromosomes in the diploid *A*. *duranensis* reference A-genome (Fig 3). Chromosome A03 comprised the largest number of DEGs (13.5%) whilst chromosome A02 had the lowest (6.9%). In accordance with previous studies [15,23], DEGs, as putative protein-coding genes, were more frequent in the distal regions of all *A*. *duranensis* chromosomes, with the gene-rich, abnormally small, chromosome A08 showing the highest density (Fig 3).

### Functional analysis

#### Functional comparison of DEGs

**Fig 4:**
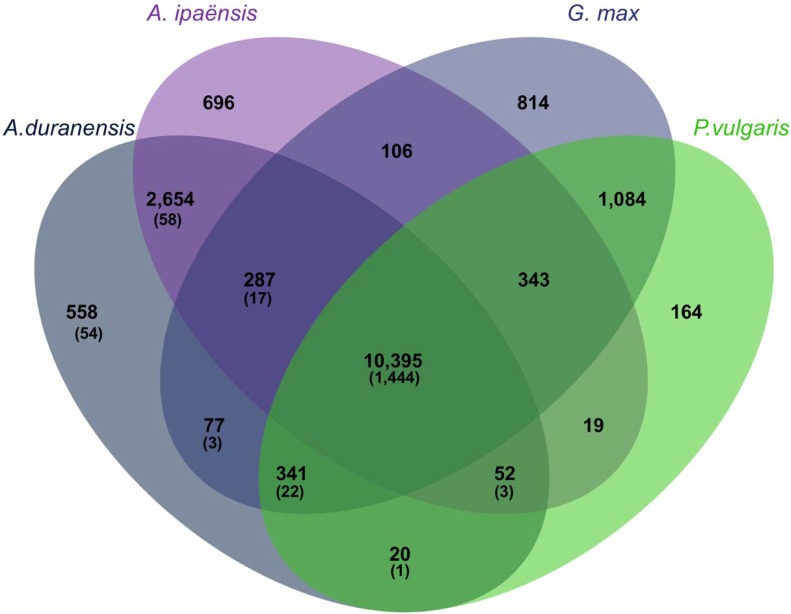
Clusters of Ortholog Groups (OGs) from four legume species. Venn diagram indicating the number of Ortholog Groups (OGs) among *Arachis duranensis; Arachis ipaënsis; Glycine max; and Phaseolus vulgaris*. The number of DEGs in the different groups is in parentheses.

A comparative analysis of the *Arachis duranensis* transcripts with their orthologs from three other legumes species (*Arachis ipaënsis*, *Glycine max*, and *Phaseolus vulgaris*) was conducted in order to identify common Ortholog Groups (OGs). *A*. *ipaënsis* and *A*. *duranensis* are closely related wild species and the most probable ancestors of the cultivated peanut [10], whilst *G*. *max* and *P*. *vulgaris* are major tropical grain legumes which, as is the case for peanut, are produced in drought-prone areas [9]. *A*. *stenosperma* was not included in this OGs analysis since the complete sequence of its genome is not yet available. A total of 17,610 OGs were identified in one or more legume species in which a large core set of proteins, consisting of 10,395 OGs (59%), were common to all four species (Fig 4). Additionally, 3,908 OGs were unique to *Arachis*, whereas only 2,062 OGs were exclusive to the non-*Arachis* species. A high number of OGs were specific to one single species, with *G*. *max* showing more exclusive OGs (814) followed by *A*. *ipaënsis* (696), *A*. *duranensis* (558), and *P*. *vulgaris* (164). The 1,602 DEGs identified in the present study as dehydration-responsive in *A*. *duranensis* and *A*. *stenosperma* (Fig 2C) were distributed within these defined clusters of legume OGs (Fig 4). In accordance with the OGs distribution, a large number of DEGs were shared among all the four species (1,444), corresponding to 90% of the DEGs, which was, surprisingly, higher than the number of DEGs common to *A*. *duranensis* and *A*. *stenosperma* (432; Fig 2C). Approximately 4% of the DEGs (58) were shared between the *Arachis* species whereas a similar number (54) were unique to *A*. *duranensis*. Consistent with the divergence of *Arachis* from the Phaseoloids sub-clade within the legume family [10], the two wild *Arachis* species showed a similar number of OGs and DEGs in common with *G*. *max* (287 and 17, respectively) and with *P*. *vulgaris* (341 and 22, respectively).

#### Functional annotation of DEGs

**Fig 5:**
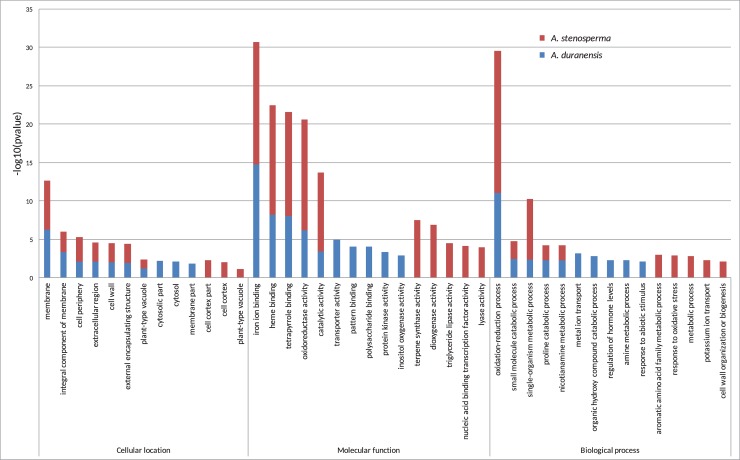
Gene ontology enrichment of *A*. *duranensis* and *A*. *stenosperma* DEGs during dehydration.

To assess the enriched Gene Ontology (GO) categories in the biological networks involved in the response to dehydration of both *Arachis* species, GO analysis was performed using the previously identified 1,602 DEGs. Although the comparison between *A*. *duranensis* and *A*. *stenosperma* showed similarities in the most abundant functional categories, important differences among the less frequent categories were found, giving insights into how each species responds specifically towards dehydration (Fig 5). Regarding cellular location, GO categories such as “membrane”, “extracellular region”, and “cell wall” showed similar levels of abundance between the two species, whereas the transcripts of the tolerant species were specific in the “cytosol” and “membrane part” categories and those from the sensitive species were specific in the “cell cortex” and “plant-type vacuole”. In the molecular function, both species had most of their transcripts similarly distributed in the “iron, heme- and tetrapyrrole binding”, and “catalytic and oxidoreductase activities” categories. Species-specific categories included “transporter, polysaccharide binding, kinase and inositol activities” for *A*. *duranensis* and “terpene, dioxygenase, lipase, transcription factor and lyase activities” for *A*. *stenosperma*. In the biological process, the majority of the transcripts were categorized under “oxidation reduction process” and some in the “proline catabolic process” both important processes in plant responses towards dehydration and drought stresses. A specific detail of the biological process categories comparison was that *A*. *duranensis* had exclusive transcripts in the “response to abiotic stimulus” category, whereas *A*. *stenosperma* had specific transcripts in the “responses to oxidative stress” category.

#### Functional analysis of Transcription Factor families

The temporal regulation of Transcription Factor (TF) expression is a key process in the downstream signaling pathways that initiate protective drought stress responses. Here, the analysis of *A*. *duranensis* and *A*. *stenosperma* transcriptome identified 142 DEGs as members of 26 TF families (S2 Fig). Of these, 66 TFs were assigned as exclusive to *A*. *duranensis*, and 26 to *A*. *stenosperma* and 50 shared between the species. Genes belonging to the bHLH (BASIC/HELIX-LOOP-HELIX), MYB (MYELOBLASTOSIS), WRKY (WRKY DOMAIN), ERF (ETHYLENE RESPONSIVE FACTOR), and NAC (NAM, ATAF, and CUC) families represented the most abundant TFs that exhibited modulations in transcript levels as part of the response to dehydration, regardless of the *Arachis* species, as previously observed [19,22,41]. Regarding species-specific TF families, seven were exclusive to *A*. *duranensis*: LBD (LOB DOMAIN), RAV (RELATED TO ABI3/VP1), DBB (DOUBLE B-BOX), ARF (AUXIN RESPONSE FACTOR), SRS (SHI RELATED SEQUENCE), SBP (SQUAMOSAL PROMOTER BINDING PROTEIN), and WOX (WUS HOMEOBOX-CONTAINING). However, only two families were found exclusively in *A*. *stenosperma*: ZF-HD (ZINC-FINGER HOMEODOMAIN) and NF-YA (NUCLEAR TRANSCRIPTION FACTOR Y SUBUNIT ALPHA) (S2 Fig). These species-specific TF-coding genes belong, in general, to novel and less-characterized classes of TFs, and some of them have been described as important regulators in the environmental stimuli and hormone responses, such as LBD, RAV, ARF, SRS, and NF-YA (http://planttfdb.cbi.pku.edu.cn/) [42,43].

### Biological pathways in response to dehydration

**Fig 6:**
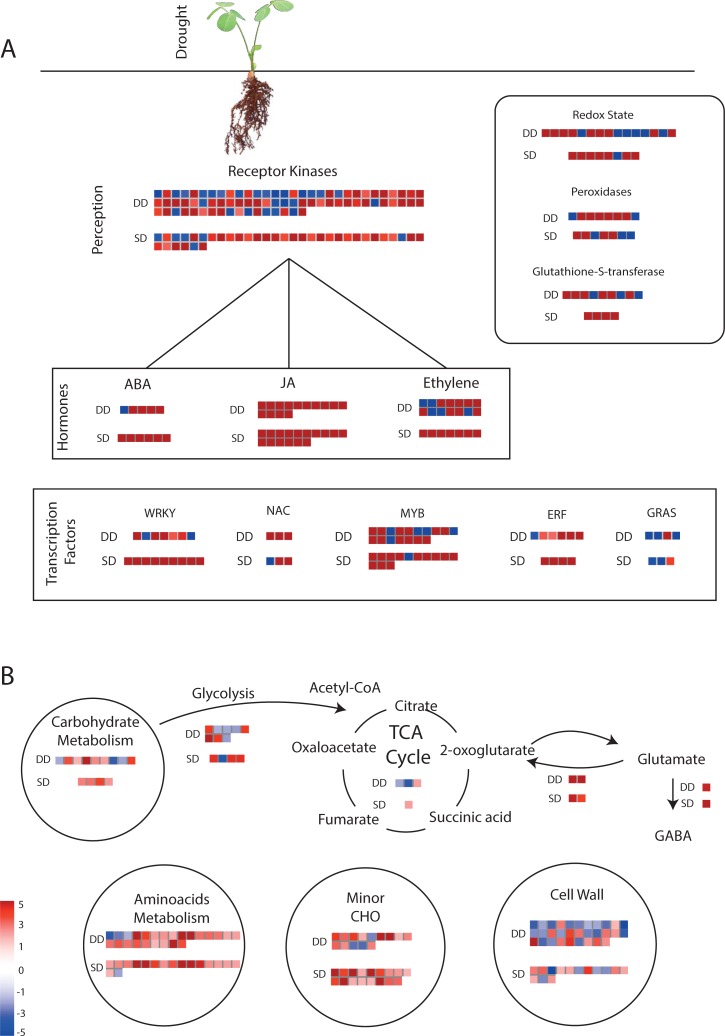
Overview of DEGs expression patterns. MapMan analysis showing molecular functional categories of DEGs expression patterns in roots of dehydration-stressed plants of *A*. *duranensis* (DD) and *A*. *stenosperma* (SD) relative to control. Squares show the different genes encoding proteins related to drought perception and oxidative responses (A) and carbohydrate and amino acid metabolism steps (B). Upregulated genes are indicated by red squares and downregulated by blue squares.

To better understand the pathways that may be involved in the differential dehydration responsiveness of the tolerant and sensitive genotypes of *Arachis* species, all 1,235 and 799 DEGs (Table 1; S5 and S6 Tables) identified in *A*. *duranensis* and *A*. *stenosperma*, respectively, were submitted to Mercator and MapMan analyses. The putative role of the products of those transcripts that were significantly up- or downregulated in response to dehydration stress was then visualized for both species (Fig 6). An overview of the general regulation patterns of genes-coding for proteins revealed the major biochemical pathways involved in dehydration responses in the tolerant and sensitive species, including hormone metabolism and signaling; regulation of transcription; oxidative stress protection; and metabolism of carbohydrates and amino acids.

In the drought perception response (Fig 6A), the tolerant genotype of *A*. *duranensis* showed a higher number and more negatively regulated genes coding for receptor kinases than the sensitive genotype of *A*. *stenosperma*. The three signaling pathways activated upon drought perception and mediated by receptor kinases [5,6] are represented in the hormones box (Fig 6A). The responses of the jasmonic acid (JA) and ABA pathways appeared similar in the two species, both in terms of quantity of transcripts and expression pattern. The ET pathway, however, showed a different response, where *A*. *duranensis* had more negatively regulated representatives than *A*. *stenosperma*. Following the signaling cascade, the overall pattern of genes coding for TFs in response to dehydration was similar in the two species, with *A*. *duranensis* exhibiting again more downregulated transcripts. The same was observed for the proteins involved in redox regulation where the drought-tolerant genotype of *A*. *duranensis* also presented more genes with a negative modulation than the drought-sensitive genotype of *A*. *stenosperma*.

In the cellular metabolism categories that were analyzed (Fig 6B), the magnitude of gene expression was generally smaller than those in the stress perception categories (Fig 6A). In carbohydrate and amino acids metabolism, overall expression behavior was similar in the two species with *A*. *duranensis* showing a higher number of regulated transcripts. In particular, in the cell wall category more than twice as many transcripts were regulated by dehydration in *A*. *duranensis* than in *A*. *stenosperma*, being the majority negatively modulated. The exception was in the minor carbohydrate category where the sensitive genotype showed more transcripts regulated than the tolerant.

An overview of the general response to “stress categories” (S3 Fig) showed that most of the DEGs were assigned to signaling, protein degradation, and secondary metabolism, whereas only three categories were exclusive to *A*. *duranensis* (hormone metabolism/salicylic acid, stress biotic receptors, and kinases) and no category was exclusive to *A*. *stenosperma*. Likewise, for metabolism (S4 Fig), 47% were assigned as exclusive to *A*. *duranensis* with none exclusive to *A*. *stenosperma*.

Based on the expression profiling revealed by Mapman analysis (Fig 6) and their functional GO categories, 20 candidate genes exhibiting differential expression behavior between *A*. *duranensis* and *A*. *stenosperma* in response to dehydration were selected for further expression analysis by qRT-PCR. These candidate genes are representative of the main pathways previously identified as being involved in drought and dehydration responses in plants, including genes acting as regulators and effectors (Fig 6). Regulator-coding candidates include: genes involved in the metabolism and signaling of the hormones ABA [*9-CIS-EPOXYCAROTENOID DIOXYGENASE* (*NCED*) and *ABSCISIC ACID 8'-HYDROXYLASE* (*ABA HYDROXYLASE*)]; ethylene [*1-AMINOCYCLOPROPANE-1-CARBOXYLATE SYNTHASE* (*ACC SYNTHASE*)]; and cytokinins [*ISOPENTENYLTRANSFERASE* (*IPT*)] or involved in signaling and transcriptional regulation as transcription factors [*DEHYDRATION-RESPONSIVE ELEMENT BINDING PROTEIN* (*DREB*), *WRKY*, and *SCARECROW*), ion transporter [*PLASMA MEMBRANE CALCIUM ATPASE* (*PMCA*)], and protein kinases (*KINASE* and *MITOGEN*). Effector-coding candidates include: genes associated with osmoregulation processes as sugar starvation [*ASPARAGINE SYNTHETASE* (*ASN SYNTHETASE*) and *FATTY ACID DESATURASE* (*FAD*)]; carbohydrate metabolism [*GALACTINOL SYNTHASE* (*GOLS*) and *PLASMA MEMBRANE MANNITOL TRANSPORTER* (*MAT*)], and cell wall biosynthesis and growth [*EXPANSIN A* (*EXPA*) and *AQUAPORIN*). In addition, other effector genes are involved in general abiotic stress responses, such as macromolecular protection [*DEHYDRIN* (*DHN*)] and senescence [*CYSTEINE PROTEASE* (*CYS PROTEASE*), *SAP*, and *F-BOX/KELCH-REPEAT PROTEIN* (*F-BOX*) were also analyzed.

### Validation of expression profile by qRT-PCR

**Fig 7:**
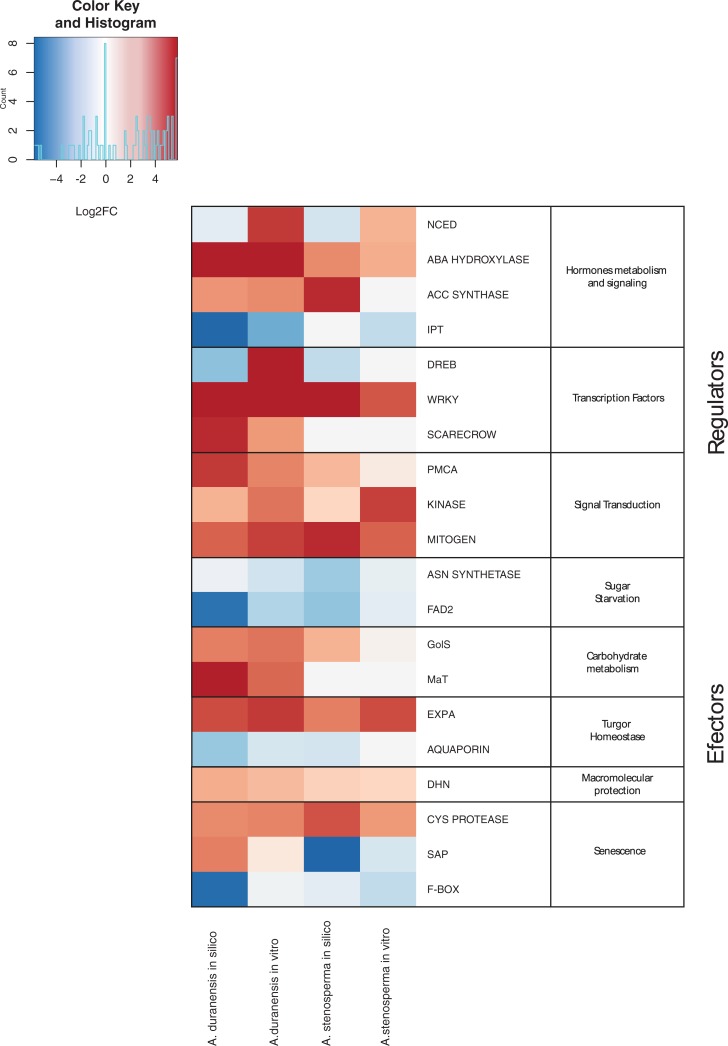
Heatmap of the relative expression of the 20 candidate genes in *Arachis duranensis* and *A*. *stenosperma* after *in silico* (RNA-Seq data) and *in vitro* (qRT-PCR) analyses. Normalized log2FC values are shown in a red–blue scale, with darker red the most upregulated and the darker blue the most downregulated DEGs.

In order to validate the RNA-Seq data, qRT-PCR analysis was carried out with the 20 selected candidate genes. All genes showed specificity of transcript amplification with high amplification efficiencies (S1 Table). Although all the primers were designed based on the sequence of *A*. *duranensis* gene-models, the majority also efficiently amplified *A*. *stenosperma* samples and produced single-peaked melting curves. The exceptions were for five genes (*ACC SYNTHASE*; *DREB*; *SCARECROW*; *MAT*; and *AQUAPORIN*) which appeared to be species-specific (Fig 7). In the case of the *SCARECROW* and *MAT* genes, they did not show significant FDR (< 0.05) when comparing the stressed to the control samples. Overall the high levels of similarity between *A*. *duranensis* and *A*. *stenosperma* orthologs emphasize the close association between AA genomes of *Arachis* [10,25]. The *in vitro* expression patterns of the 20 candidate genes estimated by qRT-PCR was consistent with those predicted by *in silico* analysis for both species using pooled CTR and STR samples (Fig 7), except for the *NCED* and *DREB* genes. Also, in accordance with the *in silico* analysis, *SAP* was the only gene which showed a different expression profile between the two species in qRT-PCR analysis, being upregulated in *A*. *duranensis* and downregulated in *A*. *stenosperma* (Fig 7). In addition to their almost identical expression behaviors, most genes presented very similar *in vitro* and *in silico* fold change magnitudes. This indicates that both *in vitro* and *in silico* analyses resulted in comparable expression profiles in wild *Arachis* and reinforced the validity of estimating gene abundance using RNA-Seq data with posterior validation using more thorough spatial and temporal qRT-PCR analysis [19,21]. Accordingly, based on the *A*. *hypogaea* expression atlas [44], weak or no basal expression of the 20 *A*. *duranensis* candidate genes homologs could be observed in non-stressed peanut roots (S8 Table). Considering that cultivated peanut (*A*. *hypogaea*) and wild *A*. *stenosperma* are more sensitive to drought than *A*. *duranensis* [24], and that the 20 selected genes showed a contrasting expression behavior between the two wild genotypes, it could be suggested that these candidates contribute, to different extents, to the tolerance of *A*. *duranensis* to dehydration stress.

**Fig 8:**
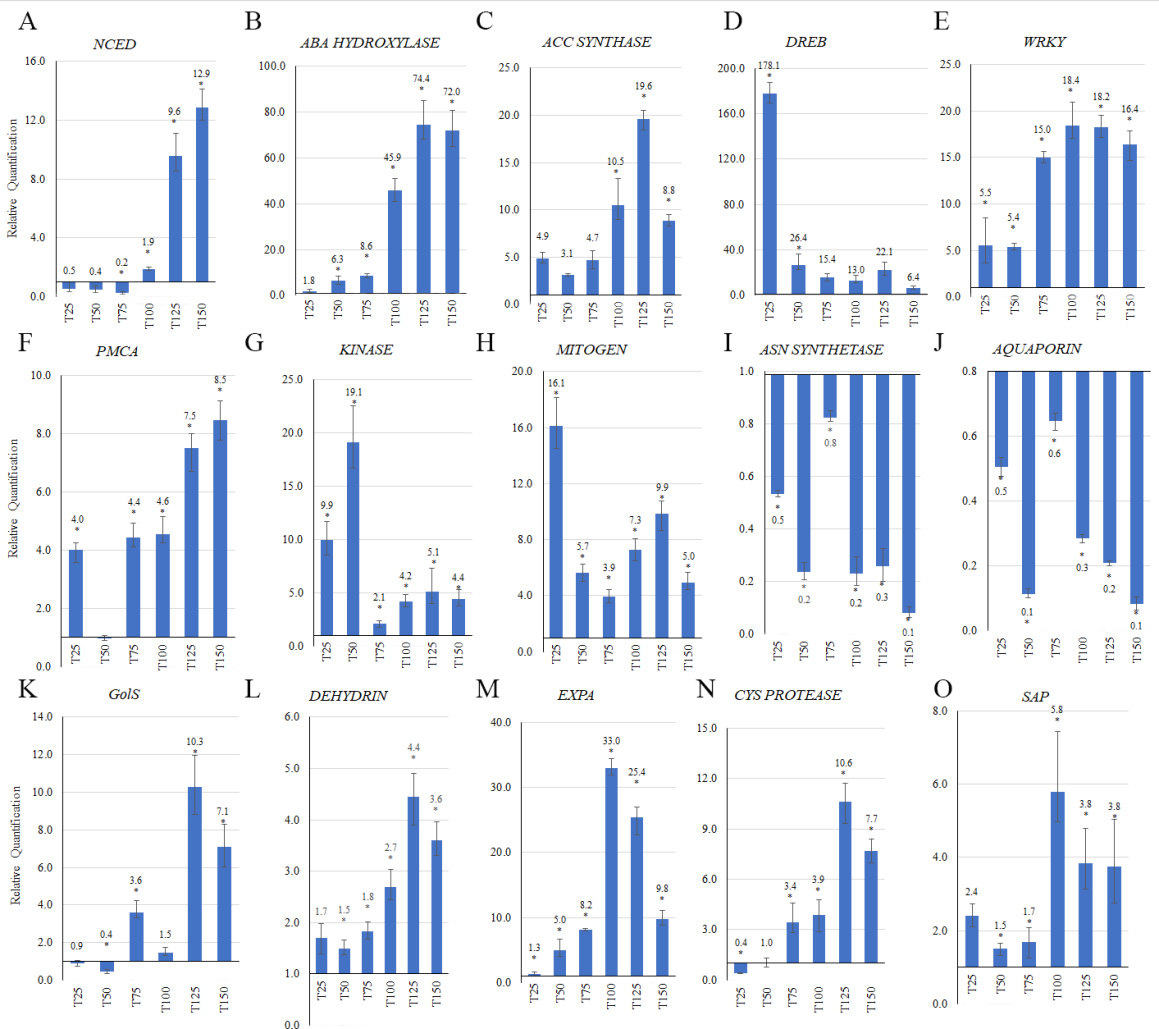
Expression of DEGs as determined by qRT-PCR. Relative quantification of mRNA levels of 15 candidate genes in *A*. *duranensis* roots during the dehydration treatment and collected after 25 (T25); 50 (T50); 75 (T75); 100 (T100); 125 (T125) and 150 (T150) min, relative to control (T0). Bars represent the standard deviation of three biological replicates. Significantly (P < 0.05) up- or downregulated genes are indicated by asterisks.

In order to track changes in expression of the candidate genes throughout the development of the dehydration stress, qRT-PCR was carried out in the tolerant *A*. *duranensis* genotype, for all time points following the beginning of the treatment, i.e., T25, T50, T75, T100, T125, and T150. In this point-by-point analysis, the expression of the majority of candidate genes in response to the dehydration was in accordance with the previous *in silico* and *in vitro* expression analyses conducted with pooled samples (Figs 7 and 8). Five candidates (*IPT*, *SCARECROW*, *FAD*, *MAT*, and *F-BOX*), for which the general expression profile did not agree with the previous results, were not considered for further analysis. The expression profiles of the three candidate genes involved in hormone metabolism and signaling (*NCED*; *ABA HYDROXYLASE* and *ACC SYNTHASE*) were quite similar, showing a gradual increase in transcript levels through the 150 min of the dehydration (Fig 8A–8C). In particular, the *ABA HYDROXYLASE* gene (Fig 8B), involved in ABA catabolism, was remarkably responsive to water deficit in *A*. *duranensis* roots, with an upregulation above 72-fold at the end of the treatment (125 and 150 min). As expected for transcriptional regulons, the two TF-coding genes were highly induced in response to the imposed dehydration stress. The *DREB* gene showed a strong upregulation of 178-fold immediately upon stress perception (25 min) followed by a rapid decline in its expression (Fig 8D), whereas the *WRKY* gene reached the peak of its expression (15-fold) at 75 min and maintained this level until the end of the treatment (Fig 8E). Similarly, other candidate genes involved in signal transduction also displayed an increase in their expression in response to dehydration, reaching the highest expression at the end (*PMCA*; Fig 8F) or the beginning (*KINASE* and *MITOGEN*; Fig 8G and 8H, respectively) of the treatment. The opposite profile, consisting of the repression of gene expression as a consequence of water withdrawal, was observed for only two genes (*ASN SYNTHETASE* and *AQUAPORIN*; Fig 8I and 8J, respectively). The remaining candidate genes, involved in general cell protection and adaptive mechanisms of drought response (*GOLS*, *DHN*, *EXPA*, *CYS PROTEASE*, and *SAP*) showed a similar overall profile, with a gradual induction as the dehydration progressed with a peak at the middle of the treatment followed by a decrease in their expression at the end (Fig 8K–8O).

## Discussion

Cultivated peanut (*A*. *hypogaea*), an allotetraploid AABB-genome, typically exhibits greater drought sensitivity than its wild diploid AA-genome relatives, possibly due to differences in morphological and physiological features relevant to drought adaptation [24]. Due to their varied levels of drought tolerance, wild relatives may offer novel genes and alleles that can be of use in the improvement of the drought tolerance of cultivated peanut [10–12,45,46]. In order to understand the multiplicity of transcriptional modulation in response to drought, two wild diploid species, *A*. *duranensis* and *A stenosperma*, were selected. These genotypes of the two species exhibit contrasting transpirational behavior, with *A*. *duranensis* showing a conservative transpiration strategy to cope with the water-limited conditions (considered tolerant) whereas *A*. *stenosperma* exhibits a less conservative transpiration behavior (considered sensitive), very close to that of the drought-sensitive peanut [24]. Changes in *Arachis* ploidy alter plant architecture, leaf morphology, and cell size, among other traits, and since these features are all involved in plant responses to drought imposition [24,47], here comparisons were made between species of the same ploidy.

In the present study, the removal of nutrient solution was used as an alternative model to simulate drought perception in *Arachis* and to facilitate rapid, uniform and easy root sampling, as previously validated in a number of different species [27,48–50]. The physiological analyses conducted at the early stages of the dehydration treatment indicated differences in leaf gas exchange between the two species, with *A*. *stenosperma* exhibiting a lower stomatal conductance than *A*. *duranensis* that is normally associated with a lower net photosynthetic rate and transpiration. This lower rate of transpiration is likely to be responsible for the slightly greater leaf temperature of *A*. *stenosperma*. A previous study reported the opposite trend for the same species [51], though it is worth noting that in that study the differences in gas exchange parameters between two *A*. *duranensis* accessions were greater than those between *A*. *duranensis* and *A*. *stenosperma*. Moreover, whilst one of the *A*. *duranensis* accessions (K7988) in that study was the same as that used here, the *A*. *stenosperma* accessions were different (accessions SV2411 and V10309), possibly also contributing to the difference in gas exchange parameters between the two studies.

The similarity between *A*. *duranensis* and *A*. *stenosperma* genomes allowed the use of the *A*. *duranensis* genome [15] as a reference to the mapping of the raw reads of *A*. *stenosperma* transcriptome data. The low difference of percentage of reads mapping between the species confirmed that they are very close in terms of genome sequence and that it is possible to use *A*. *duranensis* as a reference genome to *A*. *stenosperma*. Twenty candidate genes identified in the transcriptome of *A*. *duranensis* and *A*. *stenosperma* as differentially expressed (DEGs) in response to the dehydration treatment were selected based on both the *in silico* analyses and their putative biological function. The expression profiles of these candidates obtained by qRT-PCR corroborated *in silico* analysis. Additionally, the comparative expression analyses between the drought-tolerant genotype of *A*. *duranensis* and the sensitive genotype of *A*. *stenosperma* showed that, in general, dehydration responsiveness, including the DEGs expression magnitude, was greater in the tolerant genotype. In accordance with the physiological data, molecular evidence also indicated differences between the two species. *A*. *duranensis* exhibited, in general, a better adaptive response to water deficit at the molecular level than *A*. *stenosperma* in terms of activation of osmosensing cascades; control of hormone and osmolyte content; and protection of macromolecules. Since each candidate gene codes proteins that regulate distinct steps of biochemical pathways, putative roles in the mechanisms of receptor and effector responses and adaptation to dehydration stress in wild *Arachis* are discussed in more detail below.

### Regulator responses upon water deficit perception

Upon the perception of water deficit by plant cells, specific receptors trigger immediate molecular responses, in a process known as osmosensing. Activation of “osmosensor receptors” leads to the regulation (initiation or suppression) of a cascade of responses through signal-transducing pathways that in turn modulate the expression of genes involved in drought responses [7]. The signal sent by the osmosensor receptors is received by protein kinases (e.g., calcium-dependent kinases, CDPK, and mitogen activated kinases, MAPK) that catalyze protein phosphorylation and dephosphorylation [52]. In the present study, many receptor kinases were activated by the dehydration treatment, with differential modulation between *A*. *duranensis* and *A*. *stenosperma* (Fig 6A). Of these, two representatives of kinase-coding genes (*MITOGEN* and *KINASE*) were upregulated at all time points of the dehydration experiment. Indeed, several receptor kinases have been identified in plants subjected to water stress and shown to be involved in osmosensing process upon drought perception for the transduction of dehydration signals [7].

Besides kinases, the activation of “osmosensor receptors” upon water-deficit perception also modulates intracellular levels of calcium which in turn acts as a second messenger in a network of signalling events involved in the induction of stress responses [52]. The *PMCA* gene, coding for a calcium-ATPase present in the plasma membrane, is responsible for the active efflux of calcium from the cytosol in order to restore its low intracellular levels after the signalling events and maintain ionic homeostasis [53]. Since increases in cytosolic calcium are detrimental to plants, PMCA plays crucial regulatory roles in tolerating drought stress by activating the ABA-signalling pathway. Accordingly, we observed an induction of the *PMCA* gene under dehydration stress indicating the role of this calcium pump in the modulation of stress signalling and ionic homeostasis in *Arachis*.

Following the osmosensing process, several genes involved in ABA-dependent signaling responded to dehydration stress in this study (Fig 6), and two of the genes in this pathway were selected for point-by-point qRT-PCR analysis: *NCED* and *ABA HYDROXYLASE*. NCED catalyzes a main regulatory step in ABA synthesis by converting the epoxycarotenoid precursor to xanthoxin in the plastids that is in turn converted to ABA [54,55]. Once synthesized under water deficit conditions, ABA activates a number of ABRE-containing effector genes. Amongst several drought-related effects, ABA increases the hydraulic conductivity in root cells, directs the investment of photoassimilates towards root growth and induces stomatal closure [56]. Moreover, the overexpression of heterologous *NCED* genes in transgenic plants [56,57] demonstrated that drought tolerance could be improved through the modulation of ABA levels by this key regulatory gene. In accordance with its role in drought tolerance, in our study *NCED* gene expression levels in the tolerant genotype of *A*. *duranensis* were twice those in the sensitive genotype of *A*. *stenosperma* in response to dehydration, with a gradual increase in expression detected as the stress progressed.

The balance between biosynthesis, catabolism, conjugation and transport determines ABA content in plant organs rapidly decrease when the plants are recovering from abiotic stresses [5] and this hormone is inactivated either by conjugation or oxidation, the latter of which is catalyzed by ABA 8-HYDROLASE. Here, the *A*. *duranensis ABA HYDROLASE* gene was immediately upregulated following the beginning of the dehydration treatment and showed the highest change in expression (>72-fold) of all of the candidate genes at the end of the treatment. Previous studies in citrus plants also indicated induction of this gene under drought and combined high temperature and drought treatments and, as we also observed in *Arachis*, this induction occurred simultaneously with the induction of NCED [58]. As a result of the need for a balance between ABA synthesis and catabolism, if the expression of *NCED* is higher in the tolerant *A*. *duranensis* higher expression of *ABA HYDROLASE* might also be anticipated, as was previously observed in sheepgrass [55].

Members of many transcription factor (TF) families, including MYC, MYB, bZIP, NAC, WRKY, and DREB, are regulatory proteins involved in signal transduction and regulation of gene expression in response to different abiotic stresses in both ABA-dependent and ABA-independent cascades, that are linked by crosstalk signaling pathways [5,52,59]. The genes belonging to the DREB (Dehydration-Responsive Element Binding) TF family play an important role in the ABA-independent abiotic stress-tolerance pathways that involve the regulation of genes possessing a conserved DRE or CRT motif in their promoters [52,60]. Here, a DREB-coding gene displayed the strongest upregulation (178-fold) among candidates in *A*. *duranensis* roots at early stages of dehydration (T25), as would be expected for a TF involved in the induction of drought-tolerance responses. Additionally, heterologous overexpression of *DREB* genes from diverse origins in transgenic plants is often described as conferring increased tolerance to drought, salt, freezing, and other abiotic stresses [60].

The hormone ethylene also plays a role in modulating plant responses to abiotic stresses and acts together with ABA-signaling cascades to determine how plant organs respond to drought [6]. Recent studies indicated that ethylene signaling might be implicated in both stomatal opening and closure under stress conditions [61], The upregulation of the gene coding for ACC SYNTHASE, a key step in the production of ethylene, was observed in tolerant *A*. *duranensis* plants at all time points along the dehydration treatment. Interestingly, *Arabidopsi*s and rice mutants containing elevated ethylene levels showed increased tolerance to drought during the formation of panicles [61,62]. Together, these results suggested that ethylene production mediated by ACC SYNTHASE may be important in the determination of *A*. *duranensis* drought tolerance.

As in the case of ACC SYNTHASE, the activity of TFs belonging to the WRKY family is also modulated by both ABA- and ethylene-signaling pathways, being part of the crosstalk between these two hormones in the complex multicomponent networks of drought responses [5,63]. The WRKY-coding genes are also involved in the downstream transcriptional regulation of ABA-responsive element binding factors (ABFs/AREBs) through W-box sequences present in the promoters of the bZIP-type ABFs/AREBs TF family [63]. Here, *in silico* and *in vitro* expression analyses indicated that a *WRKY* gene was induced upon dehydration perception in both *Arachis* species, as would be expected for a regulator responsible for the induction of drought-responsive genes [5,63]. However, as for the other candidates involved in hormone signaling responses, *NCED*, *ABA HYDROXYLASE*, *PMCA*, *DREB* and *ACC SYNTHASE*, the genes in the tolerant *A*. *duranensis* had a higher magnitude of expression than their orthologs in the sensitive *A*. *stenosperma*.

### Effector responses to dehydration stress

The signaling events described above that occur upon the initial perception of water scarcity and activation of regulators ultimately lead to the expression of effector genes in *Arachis* including those associated with osmoregulation processes, macromolecular protection, and senescence (Fig 6). Under water deficit conditions, the interaction of cationic and anionic amphiphilic substances with membranes results in changes in their physical state or in the protein-lipid interactions that relay osmosensing by cells [7]. Significantly, changes in the physical state of membranes may also regulate the activity of major proteins involved in the plasticity and osmotic balance of the plasma membrane-cell wall system. Among them, Expansins and Aquaporins play essential roles in the control of cell volume and turgor homeostasis under drought stress [23,64]. In the present study, many genes coding for cell wall modifying proteins were activated in response to dehydration and an *EXPANSIN A (EXPA)* was amongst the most highly upregulated of the genes analyzed by qRT-PCR (Fig 8). These results support our previous hypothesis that wild *Arachis* Expansins are common and crucial players in responses to multiple and simultaneous stresses, including drought, ultraviolet light exposure, and nematode infection [19,21,23,65]. In contrast, *AQUAPORIN* transcripts were downregulated in all expression analyses conducted on both species. Repression of *AQUAPORIN* expression in *A*. *duranensis* after drought perception, concomitant with the induction of key enzymes in hormone biosynthesis, as observed here for NCED, ABA HYDROXYLASE and ACC SYNTHASE, agreed with previous studies showing that the inhibition of AQUAPORIN activity is highly correlated with ethylene and ABA accumulation when roots are directly exposed to water stress [64,66]. In accordance, heterologous overexpression of certain AQUAPORINS in different transgenic plants led to an enhanced ability to withstand water stress conditions [67].

In addition to the control of turgor pressure via alterations to the membrane-cell wall system under dehydration conditions, plants will also accumulate sugars and other osmolytes in order to adjust their osmotic potential. This adjustment is accompanied by the influx of water into cells, or at least reduced efflux, resulting in maintenance of the turgor necessary for cell integrity and survival [6,7,55]. In the present study, expression of many genes involved in sugar metabolism was activated during the 150 min of dehydration stress in both *Arachis* species. *GOLS* was selected as a candidate gene representative of the carbohydrate metabolism category, and, and although positively regulated in both species, showed a higher magnitude in the tolerant *A*. *duranensis*. GOLS catalyzes the formation of galactinol from UDP-galactose and *myo*-inositol and has been extensively studied as part of plant adaptive responses to handle dehydration stress. Once synthesized, galactinol is in turn used as a galactosyl donor to form the raffinose series of oligosaccharides (raffinose, stachyose, and verbascose), which may act as osmoprotectants during drought stress, but also to protect cellular membranes during seed desiccation. Indeed, there are encouraging examples of drought tolerance improvements in transgenic plants overexpressing GOLS [68,69].

As part of the overall macromolecular protection mechanisms of plants to avoid water loss during drought stress, a gene coding for an SK2-type of DHN was also selected as a candidate. DHNs have multiple protective roles in plants subject to abiotic stress, acting as chaperones, antifreeze proteins, cryoprotectants, free radical-scavengers, and ion-binding proteins in order to retain water, maintain cell turgor, and ultimately function of cellular components [70,71]. Accordingly, DHNs accumulate under various abiotic stresses that cause cell dehydration, including drought and desiccation, in processes controlled by the ABA-dependent signaling pathway. Here, a SK2-type DHN was upregulated in all analyses and both species, but again with a higher magnitude in the tolerant *A*. *duranensis* genotype. This induction was maintained during the whole dehydration experiment and could indicate that DHNs have a different role in drought adaptation in these contrasting species, as previously suggested for sheepgrass [55]. Numerous reports reveal that the overexpression of DHN-coding genes might increase plant tolerance to abiotic stresses, particularly drought, cold, and salinity, characterized by the reduction in the osmotic potential and lipid peroxidation, and accumulation of proline, sugar and potassium ions [70,71].

In addition to reduced plant growth, water deficit often leads to stomatal closure that, under severe stress conditions, may in turn lead to sugar starvation and senescence. ASPARAGINE (ASN) SYNTHETASE activity is dependent on the carbon/nitrogen status of the cell and is differently affected by various environmental stresses that may affect the requirement of asparagine for nitrogen mobilization and/or storage [72]. The regulation of plant ASN SYNTHETASE-coding genes by diverse stresses suggests convergent signal perception and transduction related to sugar-starvation conditions [73]. In our study, a decrease of *ASN SYNTHETASE* transcripts was observed in *A*. *duranensis* roots in response to dehydration stress. Since peanut is known to transport significant quantities of assimilated nitrogen in the form of asparagine [74], the decrease in *ASN SYNTHETASE* transcript levels may, therefore, reflect a prioritization under water-limited conditions of other physiological processes in wild *Arachis*, apart from nitrogen metabolism.

From the various senescence-related genes regulated during the 150 min of dehydration in both wild *Arachis* species (Fig 7) two candidates were further analyzed: those coding for a CYSTEINE (CYS) PROTEASE and a SENESCENCE-ASSOCIATED PROTEIN (SAP). CYS PROTEASEs are among the best-characterized plant proteases and known to be strongly expressed upon abiotic stress perception, being ultimately involved in programmed cell death [75]. Being a functional senescence-associated gene, CYS PROTEASE activity is normally further blocked by a protease inhibitor in drought-tolerant plants to prevent stress-related premature protein degradation. As expected, herein, a *CYS PROTEASE* gene was upregulated in both analyses and in both *Arachis* species. Accumulation of *CYS PROTEASE* transcripts was also observed in a drought-tolerant peanut cultivar under severe drought stress, whereas in the sensitive cultivar, accumulation occurred under only moderate stress [76]. Likewise, the *SAP* gene has a similar expression behavior, being induced in the drought-tolerant *A*. *duranensis* and strongly repressed in the drought-sensitive species during the imposition of dehydration. The majority of the characterized drought-induced *SAP* genes code for proteases, nucleases, and other enzymes involving nutrient recycling and stress responsive regulators, that are regulated via the ABA-dependent signaling pathway in association with other phytohormones [77]. However, most of the senescence studies refer to leaf senescence, and little is known about changes in senescent roots from a molecular, cellular and physiological perspective. Furthermore, efficient remobilization of amino acids and nutrients and the control of hydrolytic processes, by proteins such as ASN SYNTHETASE, CYS PROTEASE and SAP, could be crucial for the plant survival and reduction in water loss at whole-plant level, under water-limited conditions [6,75,77].

## Conclusions

Drought stress is a complex issue that involves an intricate chain orchestrated by physiological events affecting transcriptional and translational regulation. Different plant genotypes often harbor distinct mechanisms of response and adaptation to drought, which may include the expression of a particular set of genes that trigger changes in specific biochemical pathways. Following stress signaling, the regulators will signal towards diverse downstream pathways, which involve the action of effectors in osmoregulation, stress signaling, and macromolecule protection. In this comparative transcriptome study, many regulators and effectors were identified as being differentially regulated in two contrasting genotypes of wild *Arachis* species during dehydration. As these genes have different roles in the perception of water scarcity and subsequent establishment of stress tolerance, they might play a role in the tolerant genotype of *A*. *duranensis* earlier and/or faster response to dehydration stress in comparison with the sensitive genotype of *A*. *stenosperma*. In this manner we were able to explore the nature of drought tolerance in wild species and discover potentially useful wild alleles related to adaptability to adverse environments. The recent advances in our understanding of *Arachis* genome structure, together with functional genomic integrative approaches, has enabled new insights into gene occurrence, evolution, function, and regulation, as well as their importance in the control of desirable agronomical traits. The findings in this study will increase our understanding of the transcriptional networks that control the responses to water deficit in wild *Arachis* and will broaden the use of their extensive genetic diversity for the improvement of cultivated peanut, and other legumes, by either introgression via interspecific hybridization or transgenic approaches.

## Supporting information

S1 Fig *A*. *duranensis* 30-day-old plants at the beginning of the dehydration treatment. A) aerial part and B) roots of *A*. *duranensis* plants at T0 when the nutrient solution was removed.
(TIF)

S2 Fig Distribution of exclusive and common Transcription Factors families in *A*. *duranensis* and *A*. *stenosperma* subjected to dehydration. (EPS)

S3 Fig Number of transcripts assigned to “stress” categories of MapMan in *A*. *duranensis* (blue) and *A*. *stenosperma* (orange). (EPS)

S4 Fig Number of transcripts assigned to metabolism categories of MapMan in *A*. *duranensis* (blue) and *A*. *stenosperma* (orange). (EPS)

S1 Table Candidate genes and primers used for qRT-PCR analysis. (DOCX)

S2 Table Physiological measurements. Physiological measurements made on *A*. *duranensis* and *A*. *stenosperma* leaves during the dehydration treatment, using a portable Photosynthesis System (LI-COR, 150 model LI-6400).
(XLSX)

S3 Table Gene models identified in the transcriptome of *Arachis duranensis*. Gene models (21,125) identified from reads of *A*. *duranensis* mapped to the reference *A*. *duranensis* genome, with their respective expression values (Log2FC and FDR) and functional annotation.
(XLSX)

S4 Table Gene models identified in the transcriptome of *Arachis stenosperma*. Gene models (21,502) identified from reads of *A*. *stenosperma* mapped to the reference *A*. *duranensis* genome, with their respective expression values (Log2FC and FDR) and functional annotation.
(XLSX)

S5 Table Pairwise comparison between STR and CTR samples of *Arachis duranensis*. Differentially Expressed Genes (1,235 DEGs) in pairwise comparison between STR (stressed) and CTR (control) samples of *A*. *duranensis*, with their respective expression values (Log2FC and FDR).
(XLSX)

S6 Table Pairwise comparison between STR and CTR samples of *Arachis stenosperma*. Differentially Expressed Genes (799 DEGs) in pairwise comparison between STR (stressed) and CTR (control) samples of *A*. *stenosperma*, with their respective expression values (Log2FC and FDR).
(XLSX)

S7 Table Pairwise comparisons between samples of *Arachis duranensis* and *Arachis stenosperma*. Differentially Expressed Genes (1,602 DEGs) in pairwise comparison between samples of *Arachis duranensis* and *A*. *stenosperma*, with their respective expression values (Log2FC and FDR).
(XLSX)

S8 Table Expression of *A*. *duranensis* DEGs homologs in peanut. Expression profile of 20 *A*. *duranensis* candidate genes homologs in the *A*. *hypogaea* (cv. Tifrunner) expression atlas from the eFP Browser (http://bar.utoronto.ca/efp_arachis/cgi-bin/efpWeb.cgi).
(XLSX)
