# Supplementary figures and images for: Early responses to dehydration in contrasting wild Arachis species
Source: PLoS One. 2018 May 30;13(5):e0198191. doi: 10.1371/journal.pone.0198191 (PMC5976199; doi:10.1371/journal.pone.0198191)

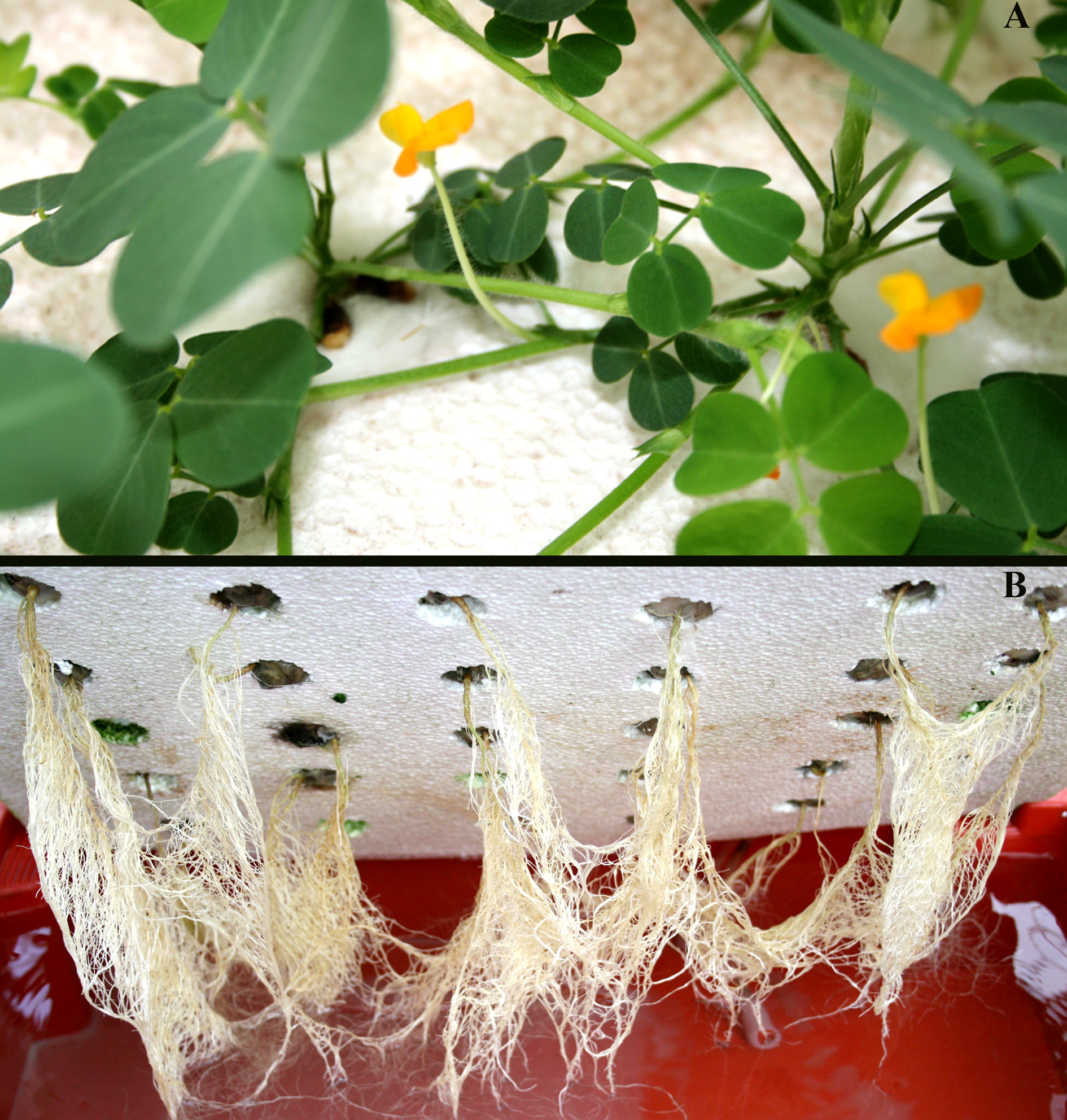

Supplement: S1 Fig — A) aerial part and B) roots of A. duranensis plants at T0 when the nutrient solution was removed. (TIF) [file pone.0198191.s001.tif]
