# Supplementary material for: Early responses to dehydration in contrasting wild Arachis species
Source: PLoS One. 2018 May 30;13(5):e0198191. doi: 10.1371/journal.pone.0198191 (PMC5976199; doi:10.1371/journal.pone.0198191)
Supplement: S1 Table — (DOCX) [file pone.0198191.s005.docx]

**S1 Table. Candidate genes and primers used for qRT-PCR analysis.**

| **Gene abbreviation** | **Gene description** | **PeanutBase ID** | **Primer sequence (5´ - 3´) Forward/Reverse** | **Amplicon size (bp)** | **Primer efficiency** |
| --- | --- | --- | --- | --- | --- |
| ABA HYDROXYLASE | Abscisic acid 8'-hydroxylase | Aradu.Q3FM7 | TTTGAGATGGCTCCAAAACC  TGAAGAGGGACAGGAAATGG | 185 | 0.900 |
| ACC SYNTHASE | 1-aminocyclopropane-1-carboxylate synthase | Aradu.62W74 | ACCATTTCGAGCCCTTTCGT  AGTTGCGGCCGAAAAATGTC | 164 | 0.866 |
| AQUAPORIN | Aquaporin TIP2-2 | Aradu.6AI81 | TTGGCTCCATTGTCGCATCT  GCCAAGTGAGCCCTTTTTGG | 176 | 0.872 |
| ASN SYNTHETASE | asparagine synthetase | Aradu.637TZ | CCCATTCAACAGCTTTCGCC  TGCTGCAAAACACGTGACTG | 191 | 0.855 |
| CYS PROTEASE | cysteine protease | Aradu.8KW68 | TCTGACGTTACGCTGCAACT  GGCCTTGCAACTCTACGACT | 178 | 0.885 |
| DHN | DeHydrin family protein | Aradu.IF4XP | TTGTCGTCAAACTCGGTGGCGA  TCATGGCAGAGGAGCACCACAA | 151 | 0.760 |
| DREB | Dehydration-responsive element-binding protein | Aradu.1ZX0E | ACCCAGTGTACAGGGGAGTG  TGCTGCCATTTCAGGTGTAG | 125 | 0.870 |
| EXPA | expansin a | Aradu.G235T | GAACATCACCAGCACCTCCA  ACCCTCCTTTGCAGCACTTT | 192 | 0.854 |
| FAD | fatty acid desaturase 2 | Aradu.7W39T | TAACGGACACACAGGAGCAG  TTTCTGAGTTCGCCGAGAGT | 187 | 0.946 |
| F-BOX | F-box/kelch-repeat protein | Aradu.WRB0S | CTGAGATGGCAAGCAGTGAA  GAGCAACTGCTCCAACATCA | 205 | 0.868 |
| GOLS | galactinol synthase | Aradu.ZK8VV | GCAGTCCATGACAGCGTAGA  ACCCAGTTTGCCATGGCTTA | 168 | 0.869 |
| IPT | isopentenyltransferase | Aradu.Z1DQ9 | CTCGGTATCTGGCTCCTCAG  AACTTGGTCTTTCCGGTTCC | 153 | 0.832 |
| KINASE | Receptor-LIKE protein kinase | Aradu.LLW1X | GGCCATGGGGTCTCTTTTGT  GCAGGCCATGAACAGCAATC | 195 | 0.858 |
| MAT | Plasma membrane mannitol transporter | Aradu.T8C1S | ATTCCTTCACTCGCCTTAGC  CAATACCTGCTGCGAGCTTT | 169 | 0.857 |
| MITOGEN | mitogen-activated protein kinase | Aradu.52QUJ | CCTGTTGGTCGAGGAAGCTT  TAGTCATCGCCAAAGCACGT | 194 | 0.842 |
| NCED | 9-cis-epoxycarotenoid dioxygenase | Aradu.Y48CE | TTTCTCCTCCCACGCATTCC  CCAGCAGGTGGTTTTCAAGC | 181 | 0.886 |
| PMCA | plasma membrane CALCIUM ATPase | Aradu.4LY04 | CCCTGGCAACACCTTATCGT  ACTGCATTGCCTCCTGCTAG | 168 | 0.855 |
| SAP | senescence-associated protein | Aradu.DSK3D | TCGGAGGGAACCAGCTACTA  GAAGGGTTCGAGTGAGAGCA | 182 | 0.858 |
| SCARECROW | scarecrow-like protein | Aradu.G1MD8 | GATGAAGGCATTCCCAAAGA  TGCATCACCTGTTGGAGAAG | 195 | 0.875 |
| WRKY | WRKY DOMAIN 25 protein | Aradu.B1C6F | TGCTCGTCCATGGAATGCCGTT  ACCGCTGGAGAAAATACGGGGA | 124 | 0.862 |
